# Sporting programs aimed at inactive population groups in the Netherlands: factors influencing their long-term sustainability in the organized sports setting

**DOI:** 10.1186/s13102-019-0137-5

**Published:** 2019-11-19

**Authors:** Linda Ooms, Mette van Kruijsbergen, Dorine Collard, Chantal Leemrijse, Cindy Veenhof

**Affiliations:** 1 Mulier Institute, PO Box 85445, 3508 AK Utrecht, the Netherlands; 2 Center of Excellence in Rehabilitation Medicine, UMC Utrecht Brain Center, University Medical Center Utrecht, Utrecht University and De Hoogstraat Rehabilitation, Rembrandtkade 10, 3583 TM Utrecht, the Netherlands; 3 Netherlands Institute for Health Services Research (NIVEL), PO Box 1568, 3500 BN Utrecht, the Netherlands; 4 Physical Therapy Research, Department of Rehabilitation, Physiotherapy Sciences & Sports, University Medical Center Utrecht, Utrecht University, PO Box 85500, 3508 GA Utrecht, the Netherlands; 5 Expertise Center Innovation of Care, Research Group Innovation of Mobility Care, University of Applied Sciences Utrecht, PO Box 12011, 3501 AA Utrecht, the Netherlands

**Keywords:** Sports club, National sports federation, Sporting program, Physical activity promotion, Inactive, Sustainability, Qualitative study

## Abstract

**Background:**

The organized sports sector has received increased interest as a setting to stimulate physical activity among inactive target groups. To include many inactive people and to obtain population health benefits, it is important that effective sporting programs are sustained (i.e. continuation of program activities) over a long period of time. This study identified the factors influencing the long-term sustainability of these kind of sporting programs located within local sports clubs in the Netherlands.

**Methods:**

Fourteen Dutch sporting programs aimed at increasing physical activity levels of inactive population groups and funded within the National Action Plan for Sport and Exercise (NAPSE) were the focus of this study. The programs were developed by ten Dutch National Sports Federations (NSFs) and implemented by different sports clubs in the Netherlands within a three-year funded implementation period (2008–2011). This research consisted of semi-structured face-to-face interviews with the program coordinators of the NSFs (*n* = 14) and semi-structured telephone interviews with representatives of sports clubs that provided the programs (*n* = 17 continued the program, *n* = 11 discontinued the program) six and a half years after the funding period ended (November 2017–March 2018). A sustainability framework with five pre-specified main themes (i.e. program design, implementation, trainer/coach, organizational setting, broader community environment) guided data collection and (deductive) thematic analysis.

**Results:**

Ten of the fourteen NAPSE funded sporting programs were sustained at the level of the NSFs. Most factors facilitating (+) and impeding (−) the long-term sustainability of the programs were common to both NSFs and sports clubs, like program adaptation (+) and a lack of program financing (−). Program evaluation (+) and high program costs (−) were specific factors mentioned by NSFs, while factors related to human resources (e.g. lack of volunteers (−)) or the sports club nature (e.g. social aspect in program design (+)) applied to sports clubs. The factors were summarized in the form of a checklist.

**Conclusions:**

Key factors influencing the long-term sustainability of the sporting programs were identified. The results can be used to develop strategies to promote long-term sustainability of these kind of programs and inform funding guidelines in countries with a similar organized sports infrastructure.

## Background

Participation in regular physical activity has positive effects on physical, mental and social health, both for children and adults [1–4]. Nonetheless, 53% of the Dutch population of 4 years and older is not sufficiently active to receive these benefits [5]. People who are insufficiently active are at increased risk for chronic diseases and mortality, especially those who are completely inactive [4, 6]. There is therefore a need for initiatives to promote physical activity among inactive target groups. In this regard, a settings-based approach to health promotion has been advocated by the World Health Organization [7]. A setting is the place or social context in which people engage in daily activities in which policy, environmental, organizational, interpersonal and personal factors interact to affect health and well-being. The settings-based approach has an ecological perspective and highlights the importance of the whole system of the setting, rather than just individual responsibility, when stimulating healthy behaviors [8–10].

Next to settings such as schools and workplaces, the organized sports sector has received increased interest by policy makers, health professionals and sport practitioners as a setting to stimulate physical activity among inactive target groups [11–16]. Sports clubs have great potential to reach many people due to their wide reach into community and the social and informal nature of participation [15, 17]. Furthermore, participation in sport at a sports club can contribute significantly to physical activity levels for health benefits [18]. Therefore, attracting inactive target groups to sports clubs seems a promising strategy to increase population levels of physical activity. However, increasing participation in sport by these inactive target groups may impose challenges as well considering the high rate of drop out among novice sport participants, the increased risk of injuries with high-intensity and competitive sports and the fact that some people do not like to participate in sport at all [19–22].

Notwithstanding these challenges, different countries have been investing resources in the organized sports sector to promote physical activity among inactive target groups. In Australia, for instance, State Sporting Organizations were funded to implement health promotion policies and practices in their associated sports clubs to create healthy and welcoming environments [12, 14]. Also, the development of cross-sectoral (including partnerships between health organizations, local governments and sports clubs) sporting programs for people who are not being active and who are on low incomes was stimulated [11, 23]. Considering the low levels of physical activity in the Dutch population, the Dutch Ministry of Health, Welfare and Sport initiated the National Action Plan for Sport and Exercise (NAPSE). The main aim of this program was: more people are sufficiently active and less people are inactive. In the program, it was stated that: ‘the range of sports and physical activities that are offered must be geared more towards encouraging inactive people and those who do not or rarely participate in sport or exercise.’ [24] Therefore, ten National Sports Federations (NSFs) received financial resources to develop sporting programs adapted to the needs and abilities of inactive people [16]. These activities had to be incorporated within the regular activities of their associated sports clubs. A total of 14 sporting programs were developed and implemented within a three-year funded implementation period (2008–2011). Most sporting programs were offered by sports clubs one ore multiple times a year during a limited time period (e.g. weeks/months) and were aimed at teaching inactive people the basics of the sport in an easy and gradual manner. Afterwards, they were encouraged to continue the sport in a beginner’s group at the sports club. An example is the six-week cycling program Start2Bike in which adults learn the basics of mountain biking or road cycling. Other sporting programs were provided by sports clubs on a continuous (i.e. weekly) basis and used simplified sport techniques and/or rules and easy to use (soft and non-threatening) sport materials to lower barriers for participation. An example is Fit Hockey, which is weekly hockey for seniors played on a small field with soft sticks and soft balls. There is evidence that these sporting programs are effective in attracting insufficiently active people and increasing their physical activity levels, but it seems a challenge to reach large numbers of the least active ones [25–27]. To include many inactive people and to obtain population health benefits, it is important that effective programs are sustained (i.e. the sports clubs’ sustainable offer of the program) over a long period of time [13]. However, many effective health promotion programs are discontinued after initial funding ends. Although a lack of financial resources after the funding period often plays a role, there may be other reasons for discontinuing programs, such as a lack of human capacity, an organization’s competing demands, an unfavorable economic environment and lack of political or community support for the program [28–31].

Different frameworks have been created to support the development of sustainable health promotion programs in health care, school and community settings [28, 30–34]. There is limited research, however, concerning the sustainability of health promotion programs implemented in the organized sports setting. One study examined factors influencing the sustainability of a funded health promotion program within sport and recreation organizations [23]. Sustainability of the health program was, for example, facilitated when the program aligned with the core values and activities of the sport organization, supportive partnerships were available and training opportunities were provided to staff. Conversely, a lack of funding opportunities was an important factor impeding sustainability of the health program. Another study, investigating the implementation period of the aforementioned NAPSE sporting programs, also found that a lack of financial resources after the funding period hindered continuation of programs, while integration of the program in the sport organization’s long-term policy facilitated program sustainability [16]. Both studies were performed directly after the funding period ended and it is not known whether programs were actually sustained over time. Furthermore, research questions were not directly examined at the level of sports clubs, but only representatives of regional and national sport organizations (i.e. Regional Sports Assemblies and NSFs) were questioned. The focus of sports clubs is mainly on providing sport activities and running competitions and they mostly rely on volunteers. Consequently, they may experience other challenges than (sport) organizations that rely on professionals or paid staff in sustaining programs that require them to work beyond their core business, such as lack of time, human resources and skills (e.g. networking skills or skills to recruit or guide inactive people) [11, 16]. Therefore, the aim of this study was to examine factors influencing the long-term sustainability of the 14 Dutch NAPSE sporting programs, considering both the perspectives of the NSFs and sports clubs, to identify similarities and differences between these two groups.

## Methods

### Definitions and framework

In the scientific literature there is little consensus on the definition of sustainability [29, 31, 34]. In this study, sustainability is defined as the continuation of a program or program activities within an organization after an initial funding period [23, 31]. There is also no consensus about how long programs or activities should be continued after an initial funding period to be called ‘sustained’, but a period of at least 2 years is frequently mentioned [29, 34]. In the current study, the focus is on factors influencing the long-term sustainability of programs, which is often conceptualized in the literature as program continuation 6 years after full implementation [35]. Due to practical reasons, the current study was performed six and a half years after the funding period ended.

The sustainability framework of Shediac-Rizkallah & Bone (1998) guided data collection and (deductive) thematic analysis [31]. This theoretical framework suggests that potential factors influencing sustainability of health promotion programs can be grouped in four main themes: 1) program design factors; 2) implementation factors; 3) factors within the organizational setting (in this study, the organizational setting of the NSF and sports club) and 4) factors in the broader community environment. This theoretical framework was complemented with other (more recent) sustainability studies [16, 23, 28–30, 32–34, 36–38]. This was done by organizing the identified factors from these studies under the four main themes by two researchers (LO, MK). However, two studies also revealed different factors related to the actual user or implementer of a program [29, 32]. In this study, this corresponded with the trainer or coach of the sports club. Therefore, these factors were included in our framework under a new and fifth main theme of factors, namely ‘trainer/coach’ (see also Additional file 1 – Theoretical framework of factors).

### Sample

This study included the 14 NAPSE sporting programs that were developed by ten Dutch NSFs and implemented by different sports clubs in the Netherlands (period June 2008–June 2011). For more information about the funded implementation period, we refer to a previous article [16]. The programs varied with regard to sport type, content, duration and target group. A description of the original programs can be found in Table 1.

**Table 1:** Description sporting programs 2011

NSF	Size NSF in 2017^a^	Sporting program	Target group 2011	Description 2011
Athletics	Large	Start to Run	Adults	6-week training program for novice runners and inactive adults aimed at running 3 km continuously. Participants train 3 times a week: one time under guidance of a professional coach, two times individually. In the final week, participants can participate in a 3 km test run. The program is offered by athletics clubs and running stores.
Judo	Medium	Judo in school	Children, adolescents	During 4–8 weeks weekly judo lessons in school provided by a qualified judo trainer, with possible follow-up lessons after school in the school location or at the judo club.
Walking	Medium	Through Four Days Marches	Adults	6-month training program for debutants of the Four Days Marches of Nijmegen. Participants can take part in the training program individually or at a walking club. From a website, training schedules and information can be downloaded. Also, questions can be asked to an (online) expert panel. Participants can take part in two walking events to prepare for the main event.
Walking	Medium	Working by Walking	Adults	Walking program of at least 16 weeks aimed at improving health parameters. The program is provided by qualified walking trainers.
Gymnastics	Large	Trendy Weeks for Masters	Older adults (45+)	During 8–12 weeks weekly gymnastics classes with a specific theme (e.g. Move on music, Winter ready) at a gymnastics club.
Hockey	Large	Fit Hockey	Older adults (50+)	Hockey played in a team with soft sticks and soft balls; training opportunities are provided on a weekly basis at the hockey club.
Swimming	Large	My Swimming Coach	Adults	A membership of the NSF, including access to an online swimming coach (including training schedules) and opportunities to participate in one-day swim clinics and events. The focus is on recreational swimmers (including adults who swim for health benefits) who swim in a swimming pool. The clinics are offered by swimming clubs and swimming pools.
Bridge	Large	Thinking and Doing	Older adults (55+)	A project of 2 years in which bridge (weekly lessons of 2,5 h) is used to create communities of older people. After a year physical activities are offered.
Sportive cycling	Medium	Cycle-Fit	Adults	6-week training program for novice cyclers (speed cycling, mountain biking) and inactive adults. Participants train 3 times a week: one time under guidance of a professional coach, two times individually. The program is offered by (sportive) cycling clubs and cycling stores.
Sportive cycling	Medium	Cycle & Enjoy Nature	Older adults (45+)	Weekly recreational cycling activities with a focus on relaxing and enjoying nature at a cycling club; or an individual introduction package including a cycling magazine, a training manual, a map with cycling routes and a calendar with cycling events.
Triathlon	Medium	Trio-Triathlon	Adults	Organization of Trio-Triathlon (the three sports of a triathlon are performed by three different individuals) events.
Volleyball	Large	Beach volleyball	Children, adolescents, adults	Organization of different beach volleyball activities (e.g. clinics, tournaments, workshops) at schools, (beach) volleyball clubs, companies and (beach) volleyball events.
Volleyball	Large	Cool Moves Volley	Children	A volleyball approach adapted to the abilities and needs of children. Training opportunities are provided on a weekly basis at volleyball clubs; clinics are provided in schools.
Volleyball	Large	Ultimate Volley Xperience	Adolescents	A volleyball event in a Caribbean atmosphere. The event is held at a special location and includes music and spectacular side-events.

*NSF* National Sports Federation

^a^Size of NSF in 2017: large > 100.000 club members; medium 25.000–100.000 club members; small < 25.000 club members

The program coordinators of the NSFs (*n* = 14), who also participated in the research concerning the implementation period [16], were invited to participate in this study by email. In case a program coordinator did not respond, a general email was sent to the NSF to get in contact with the former or current program coordinator or a representative of the NSF that was well-informed about the sporting program. In addition, the participating NSFs were asked to provide the email addresses of four sports clubs that started the program between 2008 and 2011: 1) two sports clubs that continued the program and; 2) two sports clubs that discontinued the program. In case a sports club agreed to participate, the NSF provided the email address of the sports club (i.e. of the local coordinator or trainer of the program) to the researchers. Subsequently, the sports club was invited to participate in the research by email.

### Procedures

A total of 14 semi-structured face-to-face interviews of 60–90 min duration were conducted with former (*n* = 6) and current (*n* = 8) program coordinators of the NSFs between November 2017 and March 2018. In addition, semi-structured telephone interviews were conducted with representatives of sports clubs. In case the NSF had stopped the program or no current program coordinator was present, it was more difficult to retrieve participating or non-participating sports clubs. In general, sports clubs that discontinued the program were harder to retrieve by the NSFs. Therefore, not all NSFs provided enough contact details of sports clubs and for two NAPSE sporting programs (a program that was continued by the NSF and a program that was discontinued by the NSF) no email addresses of sports clubs were provided. Consequently, a total of 28 semi-structured telephone interviews of 45–60 min duration were conducted with representatives of sports clubs (*n* = 17 continued the program, *n* = 11 discontinued the program; see also Additional file 2 - Number of interviews with sports clubs) between January 2018 and March 2018. The semi-structured interviews were performed by two researchers (LO, MK) independent of each other. The first researcher (LO) was experienced in performing semi-structured interviews. This researcher also performed the (yearly) semi-structured interviews with the program coordinators of the NSFs during the three-year funded implementation period [16]. The second researcher (MK) was instructed by the first researcher (LO) on how to conduct the interviews. Furthermore, the first two interviews were conducted by both researchers so that interviewing was done in a comparable manner. All interviews were recorded with a digital voice recorder and transcribed verbatim.

A set of predetermined open-ended questions, which were framed on the five pre-specified main themes of the sustainability framework, was used with the opportunity for the interviewer to explore particular themes or responses further. In addition, background information about the respondent, NSF/sports club and sporting program were obtained. An example of an interview guide can be found in Additional file 3 - Interview guide sports clubs continued.

The representatives of the NSFs and sports clubs were informed about the background and aims of the study through email (written) and before the start of the interview (verbally). In addition, they were informed that participation was voluntary, all collected information would be kept strictly confidential and only anonymized data would be published. All respondents provided informed consent for their participation as well as consent for their interviews to be recorded and transcribed verbatim. Participants were not exposed to procedures, nor were they obligated to follow certain behavioral rules. Therefore, in accordance with the Dutch Medical Research Involving Human Subjects Act (WMO), medical ethics committee’s approval was not necessary for conducting this study [39]. Study privacy procedures followed General Data Protection Regulations [40]. For reporting of results, the RATS (Relevancy, Appropriateness, Transparency, Soundness) guidelines were used as a guidance [41].

### Data analysis

MAXQDA version 10.0 software was used for data analysis. Data analysis was performed by the researchers (LO, MK) that were also involved in data collection. Thematic analysis was performed to examine the perspectives of the NSFs and sports cubs regarding key factors influencing the long-term sustainability of the sporting programs, highlighting the similarities and differences between the views of these two groups. For this purpose, a codebook was developed by the researchers (LO, MK) based on the aforementioned sustainability framework including the five main themes of factors (see Additional file 1 - Theoretical framework of factors). Subsequently, the following steps were taken to enhance trustworthiness of the thematic analysis as recommended by Nowell et al. (2017) [42]: First, all interview transcripts were read once by one researcher (LO) to become familiar with the data. Second, the transcripts were coded systematically by this researcher (LO), starting first with coding of the transcripts of the NSFs followed by coding of the transcripts of the sports clubs. A transcript was read again and coded using the codebook. When new subthemes emerged, new codes were inductively added. An example of a new subtheme was ‘social aspect’ - namely the social opportunities provided during the program and the social relationships acquired by participating in the sporting program. This was an important reason for (previously) inactive participants to attend and keep attending (according to trainers) and for trainers of sports clubs to continue program activities. By combining both a deductive and inductive coding approach, knowledge of previous literature and theory could be used, while simultaneously extending this knowledge with new (sub) themes derived from the raw data [43]. After initial coding, the codes (subthemes) were sorted into the five possible main themes by researcher LO. To assure quality of the coding process, a second researcher (MK) cross-checked coding of a random selection of the transcripts (19% of transcripts). In addition, the second researcher (MK) checked all derived subthemes and sorting of these subthemes in the five main themes. In case of disagreement, the results were discussed between both researchers. When no consensus could be reached, a third researcher was available (DC). However, the latter was not necessary because in all cases consensus could be reached and the discussions between the two researchers (LO, MK) only led to minor adjustments in subthemes and sorting of subthemes in main themes. After that, the coding of all transcripts was once again checked by the first researcher (LO) in the same way and order as described previously. The results of the NSFs and sports clubs were compared (by researcher LO) to identify the key (sub) themes or factors important for the long-term sustainability of a sporting program aimed at inactive people in the organized sports setting and to summarize similarities and differences between the two groups (i.e. NSFs and sports clubs). For this purpose, we compared the two groups with respect to the meaning and interpretation of (sub) themes, the frequency and prominence of (sub) themes (number of respondents mentioning a theme), and specific examples provided to support or explain a particular (sub)theme. Finally, the findings were shared and discussed with the whole research team (LO, MK, DC, CL, CV) for feedback and comment. However, this did not result in any changes in coding or (sub) themes per group.

## Results

Respondent characteristics are presented in Table 2. Although there were some changes in (local) coordinators after the funding period, most representatives of NSFs and sports clubs were involved in the sporting program for multiple years. Representatives of sports clubs were mainly the trainers that provided the program to participants. Furthermore, a diverse sample of sports clubs was represented, including sports clubs of different sizes and from all regions in the Netherlands (see also Table 2). Sports clubs that discontinued programs had provided the programs for an average of 3 years (range 1–8 years; not in Table).

**Table 2:** Descriptive characteristics of interviewees

	Program continued	Program stopped
**NSFs (n = 14)**	**(n = 10)**	**(n = 4)**
Gender (n, %)		
Female	4 (40)	1 (25)
Male	6 (60)	3 (75)
Age, mean + range (years)	43 (25–59)	50 (35–59)
Number of years employed with the NSF, mean + range (years)	14 (1/3–36)	13 (10–18)
Number of years involved in program, mean + range (years)	4 (1/4–9)	4 (2–9)
Function within NSF (n, %)		
Current program coordinator	8 (80)	0 (0)
Former program coordinator	2 (20)	4 (100)
Size NSF (n, %)		
Medium (25.000–100.000 club members)	3 (30)	3 (75)
Large (> 100.000 club members)	7 (70)	1 (25)
**Sports clubs (n = 28)**	**(n = 17)**	**(n = 11)**
Gender (n, %)		
Female	7 (41)	5 (45)
Male	10 (59)	6 (55)
Age, mean + range (years)	56 (36–78)	59 (27–79)
Number of years employed with the sports club, mean + range (years)	11 (1/6–25)	24 (5–52)
Number of years involved in program, mean + range (years)	8 (2–15)	3 (1–8)
Function within sports club (n, %)^a^		
Trainer/coach	12 (71)	8 (73)
Board member	4 (24)	0 (0)
Chairperson	2 (12)	2 (18)
Secretary	1 (6)	2 (18)
Other	0 (0)	2 (18)
Size sports club (n, %)		
Small (≤ 100 club members)	3 (18)	1 (9)
Medium (101–300 club members)	7 (41)	6 (55)
Large (≥ 301 club members)	6 (35)	2 (18)
Unknown	1 (6)	2 (18)
Region in the Netherlands (n, %)		
North	4 (24)	1 (9)
East	6 (35)	2 (18)
West	4 (24)	4 (36)
South	3 (18)	4 (36)

*NSFs* National Sports Federations

^a^An interviewee could have multiple functions within the sports club

Table 3 describes continuation of and changes made to the sporting programs after the funding period by the NSFs. Six and a half years after the funding period ended, ten of the fourteen NAPSE funded sporting programs were sustained at the level of the NSFs, but one program without direct involvement of sports clubs. Previously, people could participate in this program on an individual basis or at a sports club, but the NSF only continued the individual training option. Many sports clubs, however, still provided their own training programs for inactive people or people novice to the sport. For eight of the sustained programs, a NSF program coordinator was present and nine NSFs still provided support (e.g. materials, advice) to their associated sports clubs to run the programs. During the years, some changes were made in target group (*n* = 6 programs) and/or content (*n* = 9 programs) of the programs. The other four NAPSE funded sporting programs were directly stopped at the level of the NSF after the funding period. However, one program was still offered by sports clubs without involvement of the NSF. For another program, coordination of the NSF was not necessary anymore, because sports clubs (and event organizations) could run the program on their own.

**Table 3:** Continuation of and changes made to sporting programs by NSFs (2017–2018)

Sporting program	Continued by NSF	NSF program coordinator	Support of NSF to sports clubs (M, K, F, T)^a^	Changes in target group or content after the funding period?
Yakult Start to Run (previously: Start to Run)	Yes.	Yes.	Yes (M, K, F, T).	*Target group*: No. *Content*: Yes, the duration of the program is now 7 weeks with the test run in week 7. Sometimes, local sports clubs replace the individual training sessions for more guided training sessions. Furthermore, there is a running app (audio coaching) that participants can use for the individual training sessions. Also, the name of the program has changed into Yakult Start to Run. Yakult is the main sponsor of the sporting program and plays an important role in promotion of the program.
Judo in school	Yes.	No.	Yes (M, K).	*Target group*: Yes, the focus is predominantly on children. *Content*: Yes, judo lessons in school vary from 1 to 8 lessons. Follow-up lessons are mostly at the club.
Through Four Days Marches	Yes, but people can only take part in de training program individually. The NSF does not collaborate with walking clubs anymore for the training program. However, walking clubs still offer their own training programs.	Yes.	No.	*Target group*: No. *Content*: Yes, the individual training schedules have been improved including interval, power and technical training. Participants can take part in a regional meeting where they get information about their training schedule and the Four Days Marches of Nijmegen (e.g. how to train well, which clothes and shoes to wear). In addition, they can take part in one preparatory walking event. The regional meetings are organized by walking trainers. Participants can only take part in the training program individually. There is no direct link anymore between the national training program and the local training programs that walking clubs (still) offer themselves.
Working by Walking	No.	N.A.	N.A.	N.A. The NSF did develop a new program ‘Walking Fit’ using elements of the old program.
Flexible (previously: Trendy Weeks for Masters)	Yes, but the changed program has not been implemented yet.	No.	Yes (M, K, T).	*Target group*: No. *Content*: Yes, the original themes (e.g. Move on music) are not used anymore. The 8–12 weeks gymnastics classes focus now on condition, power, flexibility and coordination. For example, all flexibility exercises from all old themes are now combined into the theme ‘flexibility’. Also, a trainer course has been developed for the changed program. Finally, the name of the program has been changed into ‘Flexible’ due to confusion about the term ‘Masters’ (which age groups belong to the Masters category?).
Fit Hockey	Yes.	Yes.	Yes (M, K).	*Target group*: No. *Content*: No.
My Swimming Coach	Yes.	Yes.	Yes (M, K, T).	*Target group*: Yes, the focus is next to recreational swimmers (including adults who swim for health benefits) who swim in a swimming pool also on competitive swimmers and people who swim in open water. *Content*: Yes, the training schedules and one-day clinics have been adapted to the target groups. Also, the program is expanded with training courses of multiple weeks (e.g. front crawl course).
Thinking and Doing	Yes.	Yes.	Yes (M, K).	*Target group*: Yes, the focus is on adults 60+ years. *Content*: Yes, the bridge course material is adapted to the older target group (60+). The physical activity component is now an optional component.
Start2Bike (previously: Cycle-Fit)	Yes.	Yes.	Yes (M, K, F, T).	*Target group*: Yes, the focus is more on novice cyclers and less on inactive people. *Content*: Yes, instead of 6 training sessions of 2 h, the program consists of 4 training sessions of 3 h (the program duration is 4 weeks). The training content itself is the same. Also, the name of the program has changed into Start2Bike, because this name reflects more the learning of cycling techniques.
Cycle & Enjoy Nature	No, but sportive cycling clubs still offer the program.	N.A.	N.A.	N.A.
Trio-Triathlon	No, because local event organizations/clubs can organize the Trio-Triathlon events themselves now and also do this.	N.A.	Yes (K).^b^	N.A.
Beach volleyball	Yes.	Yes.	Yes (M, K, T).	*Target group*: Yes, the focus is more on adolescents and (young) adults. *Content*: Yes, in addition to the existing activities the NSF also organizes the Beachvolleyball circuit (15 weekends from May to August with tournaments). Also, new one-day events are developed, like the ‘Join Volleyball bus’, which visits schools. This bus includes all kinds of (beach volleyball) materials so that people can become acquainted with the sport.
Cool Moves Volley	Yes.	Yes.	Yes (M, K, T).	*Target group*: Yes, first the age group was children from 5 to 12 years. Now the age group is children from 6 to 12 years. The activities were too difficult for children 5 years of age. *Content*: Yes, there are minor changes in the game rules and technical exercises. Furthermore, Cool Moves Volley is now the official volleyball form to teach children 6 to 12 years the fundamentals of volleyball at volleyball clubs and also the official competition form for this age group.
Ultimate Volley Xperience	No.	N.A	N.A.	N.A. An element of de program (side-event) is continued by a commercial organization.

*N.A* not applicable, *NSF* National Sports Federation

^a^Support of NSF to sports clubs: *M* materials (e.g. promotional materials, trainer manual), *K* knowledge/advice, *F* financial resources, *T* trainer courses specific for the program

^b^Local organizations and clubs can still contact the NSF for advice when they want to organize a Trio-Triathlon event

The sustained programs varied widely with regard to the number of activities offered per year, their actual reach and the number of participants becoming a member of a sports club/the NSF (see Table 4). When there was no NSF program coordinator or a program was completely stopped at the level of the NSF (but not at the level of sports clubs), this information was often unknown.

**Table 4:** (Sustained) sporting programs: number of activities, involved locations, participants and membership

Sporting program	Number of activities per year	Locations in 2017–2018 (n)	Average number of participants per year (n)	Average percentage of participants that becomes member of a sports club/the NSF (%)
Yakult Start to Run (previously: Start to Run)	The 7-week training program is offered two times a year in March and September in different locations.	180 (171 athletics clubs, 9 other locations)	3.500	90% of participants wants to continue running and of these 84% wants to do this at an athletics club.
Judo in school	Unknown^a^	100 judo clubs	Unknown^a^	Unknown^a^
Through Four Days Marches	The individual training program is offered one time a year for a period of 6 months with weekly training schedules, a start meeting and participation in a preparatory walking event.	N.A.	1.500	80% of participants is still member of the NSF 1 year after finishing the program.
Working by Walking	N.A. Both the NSF as well as walking clubs stopped offering the program.	N.A.	N.A.	N.A.
Flexible (previously: Trendy Weeks for Masters)	Unknown^a^	Unknown^a^	Unknown^a^	Unknown^a^
Fit Hockey	The program is offered on a weekly basis at hockey clubs during the whole year.	20 hockey clubs	160	100%, all participants are member of a hockey club/the NSF.
My Swimming Coach	Online platform with training schedules for the whole year; 6 one-day swim clinics; the 12-week front crawl course is offered 3 times a year in different locations.	90 swimming pools with 54 involved swimming clubs	3.000	100%, all participants are member of the NSF. It is unknown how many participants become member of a swimming club.
Thinking and Doing	The 2-year program is offered two times a year in two different locations/municipalities.	2 locations/municipalities	120	35% of participants becomes member of a bridge club/the NSF; another 40% of participants continues to play bridge, but only with others at home.
Start2Bike (previously: Cycle-Fit)	The 4-week training program is offered two times a year in April and October in different locations.	100 sportive cycling clubs, some in collaboration with cycling stores	1500	30% of participants becomes member of a sportive cycling club/the NSF.
Cycle & Enjoy Nature	The NSF does not offer the program anymore, but sportive cycling clubs still offer the program on a weekly basis from April until October.	Unknown^b^	Unknown^b^	Unknown^b^
Trio-Triathlon	The NSF stopped coordinating the events. Local organizations/clubs organize the events. A Trio-Triathlon event is organized one time a year by an organization/club.	101 Trio-Triathlon events in different locations	7.890	Unknown^b^
Beach volleyball	2.000 Beach volleyball clinics in schools and companies; one time a year the Beach volleyball circuit (15 weekends from May to August); 300 other one-day events at schools.	300 locations with structural Beach volleyball activities (e.g. Beach volleyball clubs, beach accommodations)	63.500 (50.000 clinics, 10.000 Beach volleyball circuit, 3.500 one-day events)	Unknown
Cool Moves Volley	The program is offered on a weekly basis at volleyball clubs during the whole year. Also, the program is part of physical education classes (PE) in primary schools. In addition, 2.000 clinics are offered at primary schools.	4.434 (350 volleyball clubs and 4.084 primary schools with CMV activities during PE)	51.000 (16.000 CMV-members, 35.000 participants of clinics). It is not known how many children are reached during PE classes.	100%, all participants at volleyball clubs are member of the club/the NSF. It is not known how many participants of clinics become member of a volleyball club/the NSF.
Ultimate Volley Xperience	N.A. Both the NSF as well as volleyball clubs stopped offering the program.	N.A.	N.A.	N.A.

*N.A* not applicable, *NSF* National Sports Federation

^a^Unknown, due to lack of central coordination by the NSF and/or absence of a NSF program coordinator

^b^Unknown, because at the level of the NSF the program has stopped completely

### Key factors influencing the long-term sustainability of sporting programs aimed at inactive population groups

In Table 5, the key facilitating (+) and impeding factors (−) influencing the long-term sustainability of the sporting programs are presented per main theme. Inductively added factors are presented in bold. Most factors were mentioned by both NSFs and sports clubs (*n* = 17, e.g. *Program design*: program adaptation (+)). However, some factors were more important to NSFs (*n* = 5, e.g. *Implementation*: program evaluation (+)) or sports clubs (*n* = 9, e.g. *Program design*: social aspect (+)). The results are described in more detail below under the five main themes - program design, implementation, trainer/coach, organizational setting NSF/sports club and broader community environment. Since the impeding factors were often the inverse of the facilitating factors, they are not always explained separately. Factors common to both NSFs and sports clubs are described first (*common factors*), followed by description of factors specific to NSFs or sports clubs (*specific factors*).

**Table 5:** Key factors influencing long-term sustainability of sporting programs aimed at inactive target groups^a^

Main theme	Factors important to both NFSs and Sports clubs *(common factors)*	Factors important to NSFs *(specific factors NSFs)*	Factors important to sports clubs *(specific factors sports clubs)*
*1. Program design*	**• Program alignment with needs inactive target group (+)** **• Become acquainted with the sport in an easy and right way (+)** **•** Program adaptation (+) **• Program does not align with needs inactive target group (−)**	**• High program costs (−)**	**• Social aspect (+)** **• Fun (+)**
*2. Implementation*	• Training and education (+) • Positive program effects (+) **• Recruitment inactive target group is difficult (−)**	• Program evaluation (+)	**• Low participant numbers (−)**
*3. Trainer/coach*	**•** Knowledge trainer (+) **• Personal approach of participants (+)**		
*4. Organizational setting NSF/sports club*	• Compatibility of program with organization’s mission/activities (+) • Benefits (+) • Program financing (+) **• Support of NSF to sports club (+)** • No compatibility of program with organization’s mission/activities (−) • No benefits (−) • Lack of program financing (−)	• Program coordinator (+) • Absence or leave of program coordinator (−)	**• Enthusiastic and committed leader (+)** **• Availability of professional trainers (+)** **• Absence or leave of enthusiastic and committed leader (−)** **• Lack of/dependence on volunteers (−)** **• No support of NSF to sports club (−)**
*5. Broader community environment*	• Partnerships (+)	**• Popularity sport/program (+)**	**• Competing programs/activities (−)**

*NSFs* National Sports Federations

^a^Inductively added factors are presented in **bold**; (+) facilitating factor for sustainability; (−) impeding factor for sustainability

### Program design

#### Common factors

NSFs and sports clubs continued sporting programs because they aligned with the inactive target group’s needs. For inactive people, the threshold to participate in sport in general or at a sports club in particular may often be too high. The sporting programs provided opportunities for this target group to become acquainted with the sport in an easy and appropriate manner without getting injuries. Afterwards, these people are experienced enough to continue the sport in a beginner’s group at the club.

Program adaptation was another program design factor enhancing programs’ long-term sustainability. During the years, sporting programs were adapted (i.e. with regard to content or organizational aspects) both by NSFs and sports clubs, to constantly meet the needs of the (previously) inactive target group and the sports clubs. Continuous sporting programs were, for instance, (gradually) adapted to the (previously inactive) participants becoming more physically active (e.g. creating a beginner’s and more advanced group). Changes were also made to programs due to the availability of new knowledge or technologies (e.g. including more strength and flexibility exercises in training sessions to prevent injuries with inactive people, using a running app instead of a training schedule per email), new partnerships (e.g. changing program name in sponsor name) and sometimes decreased financial resources (e.g. providing less training sessions in schools). Mostly, only minor adaptations were made (see also Table 3):

*“We changed the program from six training sessions of 2 hours to four training sessions of 3 hours, based on feedback of participants. First, they had to plan six weekends free, now only four weekends. And with 2 hour sessions, there was actually little time left to cycle, because you had to move to the starting location of the training and due to the time needed to startup the training. So now they have more time to cycle.” (NSF, continued program)*

#### Specific factors

(Trainers of) sports clubs continued sport activities due to their social and fun characteristics: The social opportunities provided during the program (e.g. drinking coffee/tea before, during or after training, going to and participating in a sport event together), the acquired social relationships and having fun during participation were main reasons for (previously) inactive sport participants to attend and keep attending the programs (according to trainers) and also for trainers to stay motivated themselves.

High program costs (e.g. due to the use of expensive program materials, intensive guidance of participants or the need to rent a specific (sports) accommodation), which could not be financed from participant fees alone and for which external financial resources were needed, resulted in discontinuation of programs by NSFs.

### Implementation

Key implementation factors influencing the long-term sustainability of programs are described below. It should be noted that these factors were not only important during the three-year funded implementation period but also thereafter.

#### Common factors

NSFs (e.g. national or regional trainer courses) and sports clubs (e.g. trainers transmitting knowledge and skills to other trainers) provided trainer courses specific for the sporting programs on a regular basis (i.e. once or multiple times a year). These were very important for sustaining the programs: they provided trainers of sports clubs with the necessary skills to guide the inactive target group and assured sufficient (professional) trainers were available.

Another implementation factor facilitating the long-term sustainability of programs was their effectiveness. When a program showed positive effects with participants, like participants learning the sport and becoming more physically active, NSFs and sports clubs were more likely to continue it. Also, partners and sponsors were more willing to contribute (and keep contributing) to effective programs:

*“The schoolteachers think the sport lessons are very important. They see the positive effects of the lessons on the behavior of the children and how they interact with each other. So we keep continuing the lessons. We bought training suits together.” (Sports club, continued program)*

The sporting programs were aimed at increasing participation in sport by inactive target groups. The NSFs promoted their programs via national press, the internet (including social media), television and partner organizations. The actual recruitment of participants was done locally by sports clubs through the distribution of posters, flyers and leaflets. Also, adverts were placed in local newspapers and on social media and participants were recruited by word of mouth. When sufficient participants were recruited, a large part was often already somewhat physically active at the start. In general, it was difficult to recruit large numbers of inactive people through these standard recruitment strategies and trainers of sports clubs did not always have the knowledge or resources to get in contact with the inactive target group. This also resulted in discontinuation of programs:

*“The inactive and overweight target group was difficult to reach. We underestimated this. To get in contact with this target group you need more. You need other expertise and contacts next to a sport trainer.” (NSF, discontinued program)*

It should be noted, however, that some NSFs and sports clubs did manage to reach larger numbers of inactive people through collaboration with organizations or people that are close to this target group (e.g. organization for older adults, physiotherapist, general practitioner). These organizations or people promoted the sporting program to inactive people or referred inactive people to the programs (see also factors *Broader community environment*).

#### Specific factors

Program evaluation contributes to the long-term sustainability of programs according to NSFs. It was done by the NSFs to demonstrate program effectiveness and to ensure the program fitted with the target group and sports clubs. NSFs used different methods for evaluation, like (online) questionnaires, interviews and group meetings with participants and/or trainers:

*“Every year, we evaluate with all 70 involved trainers. We ask for their opinions: the opportunities they see, the barriers they experience. What do they notice with the target group? They are close to the participants, so they are well-informed about their experiences. Also, participants receive an online questionnaire. Based on the results, we give feedback to the trainers, so that they can further develop themselves. We also use the results to improve the courses and clinics.” (NSF, continued program)*

For sports clubs, low participant numbers (also in relation to the difficulty to recruit large numbers of inactive people) was an important factor hindering sustainability of their sporting programs.

### Trainer/coach

#### Common factors

The sporting programs aimed to encourage (constantly new) inactive people to participate in sport. Both NSFs and sports clubs agreed that for their long-term sustainability it is very important that the appointed trainers have the knowledge and skills to guide this particular target group. This ensures that participants have positive sport experiences and gladly come back to the club to participate in (additional) sport activities. In this regard, a personal approach of participants is desired:

*“The person in front of the group is very important. Participants want to have the feeling ‘he (the trainer) sees me’, ‘he knows what I am doing’.” (Sports club, continued program)*

As mentioned previously, providing training and education opportunities to trainers is a way to realize this (see *Implementation* factors).

#### Specific factors

There were no trainer/coach factors specific to NSFs or sports clubs.

### Organizational setting NSF/sports club

Most retrieved factors important for the long-term sustainability of programs were related to the organizational setting of the NSFs and sports clubs.

#### Common factors

Sustained programs aligned with the NSFs’ and sports clubs’ core values and activities:

*“This program fits with the DNA of our organization. It’s the DNA of our sport. There will always be a concept like this within our organization.” (NSF, continued program)*

Programs were also continued by NSFs and sports clubs due to the acquired benefits, such as more people becoming familiar with the sport or sports club, a better image of the sport, attracting new target groups, more participants, new club members and people who are willing to do club volunteer tasks. However, programs were stopped when they did not align with the organizations’ core values and activities and program benefits were absent:

*“We are not the right organization to do something with this target group. The program has no advantages for us. It even has no societal advantages.” (NSF, discontinued program)*

Sustained programs secured their financial resources. For both NSFs and sports clubs this included internal financial resources, participant/membership fees and in some cases sponsorship fees. Financial resources were not only used to run the programs, but also to educate trainers, to promote the program, to buy (sport) materials and to further develop the program. On the other hand, a lack of program financing, sometimes in combination with high program costs (see *Program design* factors), was an important reason for NSFs and sports clubs to discontinue programs.

Most NSFs still supported sports clubs in different ways, for example, by providing them with knowledge and advice, (promotional) materials, financial resources and training and education opportunities (see also Table 3). Promoting the sporting programs nationally and supporting clubs with organizational aspects were other examples of NSF support to clubs. This saved sports clubs a lot of time and made it possible for them to focus mainly on the sport activities and guidance of participants. Both NSFs and sports clubs, therefore, agreed that the support that NSFs offered was of great value to sports clubs for continuing activities.

#### Specific factors

For some sports clubs, a lack of support of their NSF was a reason why they stopped offering programs:

*“First, the NSF had three club advisors, but they left due to cut downs. After that, we were not approached anymore to implement the program. The club advisors always helped us. They made the connections with municipalities and schools. We implemented the program multiple times, but now we have stopped. I think because we are not approached anymore.” (Sports club, discontinued program)*

The remaining (specific) organizational factors influencing long-term sustainability were related to human resources. Having one or more persons in the organization responsible for coordinating the program within the organization facilitated the long-term sustainability of programs. On the level of the NSF, this was the employed NSF program coordinator, sometimes assisted by other NSF employees. This person focused, for instance, on (coordinating) national promotion of the program, recruitment and assistance of clubs and recruitment of partners. Sports clubs relied on one or more enthusiastic committed trainers or volunteers who were occupied with the recruitment of (other) trainers and participants and all kinds of other organizational aspects:

*“I am the head trainer and I coordinate the program. There are eight assistant-trainers and two other educated trainers. I coordinate all actions and assign tasks to everyone. Every two months, we meet with all trainers to discuss everything, like problems encountered during implementation or problems with participants and so on. The trainers alternate during the training sessions. We do this with a lot of enthusiasm. We like to be in front of a group of participants and to share our enthusiasm for the sport.” (Sports club, continued program)*

Programs were less sustainable when there was no national coordinator present. Also, continuation of programs was threatened when the national coordinator would leave. The same was true for sports clubs regarding their local coordinator. For sports clubs, it was even more difficult to find a (new) coordinator due to their reliance on and often a lack of volunteers:

*“Well, I am doing this now for the third or fourth year. One day, I will look around to see whether someone could take over my task. When there is no one who wants to do this, this could impede continuation of the program. It is common in volunteering that people want to help and support, but it is sometimes difficult to find a real coordinator or leader.” (Sports club, continued program)*

With regard to human resources, sports clubs also mentioned the availability of (enough) professional trainers as an important factor for continuing activities at the sports club level. As described previously, professional trainers enhanced sport experiences of (inactive) participants and, in this way, contributed to continuation of programs (see also factors related to *Implementation* and *Trainer/coach*).

### Broader community environment

Several factors in the broader community environment were reported that influenced the long-term sustainability of programs.

#### Common factors

Long-term partnerships were important for sustaining programs, both at the national and local level. NSFs collaborated, for instance, with (sport) event organizers, municipalities, sport stores and other commercial organizations (e.g. from food and drink industry). Local sports clubs collaborated amongst others with schools, municipalities, organizations for older people, sport stores, health professionals (e.g. a general practitioner, physiotherapist) and other sports clubs. Partners promoted the programs, supported in recruitment of inactive participants (e.g. referral of inactive people to program), provided financial, material or human resources and shared their expertise or facilities. Brand awareness, more participants (e.g. for sport events), attracting new customers (e.g. for sport stores) and contributing to more healthy or physically active people were examples of benefits for partner organizations.

#### Specific factors

The popularity of a sport in general or of the sporting program in particular supported it’s long-term sustainability according to the NSFs:

*“Many people do the sport and the sport is still growing enormously. We encourage people to practice it with this program. We show people that practicing the sport in a safe and appropriate manner is important to us.” (NSF, continued program)*

On the other hand, competing programs or sport activities threatened continuation of sporting programs according to sports clubs:

*“In our municipality, many children play soccer and handball. And there is hockey and they offer all kinds of other sports. Children have many options to choose from which leaves fewer children for our activities.” (Sports club, continued program)*

### Results in the ecological perspective

**Fig. 1:**
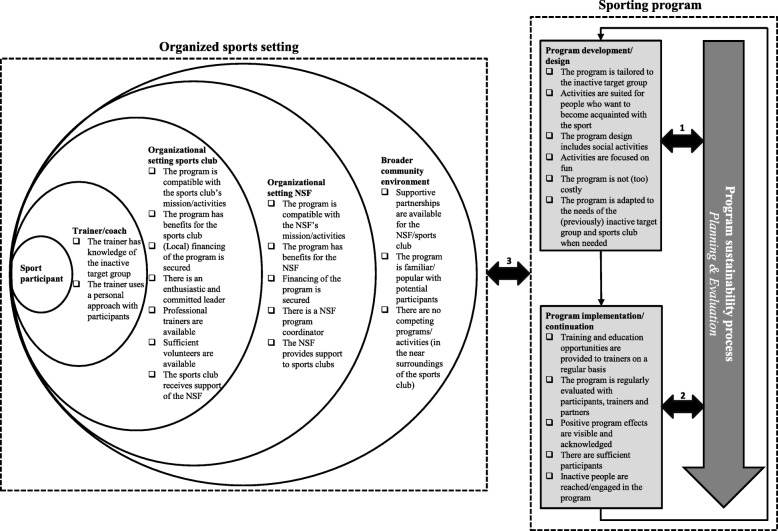
Results in the ecological perspective. *NSF* National Sports Federation. The organized sports setting and the different levels of influence - broader community environment (policy and environmental level), organizational setting NSF, organizational setting sports club (organizational level), trainer/coach (interpersonal level) and sport participant (personal level) - are presented on the left. The sporting program and it’s different phases - program development/design and program implementation/continuation - are presented on the right

Considering the ecological perspective of the settings-based approach to health promotion [8–10], an ecological model is used to summarize the results. The factors are presented in the form of a checklist, which can be used as guidance to enhance the long-term sustainability of sporting programs aimed at (previously) inactive target groups and implemented in the organized sports setting (see Fig. 1). Figure 1 illustrates that the long-term sustainability of a program is a continuous process: it should be considered from the very beginning, i.e. during program development/design *(arrow 1)*. Furthermore, continuous attention should be paid to sustainability during the implementation/continuation phases (*arrow 2*). Moreover, the long-term sustainability of a sporting program is influenced by all levels of the organized sports setting (*arrow 3*), either directly or indirectly through program development/design and program implementation/continuation factors (e.g. sport participant level). By taking into account the different factors that influence long-term sustainability, the sustainability process in turn influences program development/design, program implementation/continuation and the different levels of the organized sports setting. This is visualized by reciprocal arrows (*arrows 1–3*).

## Discussion

### General findings

This study identified factors influencing the long-term sustainability of the 14 Dutch NAPSE sporting programs aimed at inactive people, taking into account both the views of the NSFs and sports clubs. It is positive that ten of the fourteen NAPSE sporting programs were continued by the NSFs (and their associated sports clubs) six and half years after the funding period ended. Programs were sustained in different ways, for instance, with and without coordination or support of the NSF, continuing all or a part of the activities and with varying reach into the community.

Although financial resources were important for NSFs and sports clubs to sustain programs, there were many other common influencing factors, such as program adaptation and program effectiveness. However, there were also differences in influencing factors between NSFs and sports clubs, which were specific to the context of these organizations, i.e. a professional-led organization vs. a mostly voluntary-based organization (see also Table 5 and *Comparison of factors with other studies*).

Furthermore, the long-term sustainability of a sporting program is a continuous process, which starts at program development and continues through program implementation and continuation (after a funding period) phases. Therefore, early and active planning is needed to create the conditions that enhance the long-term sustainability of programs. This requires formulating sustainability objectives (e.g. what is to be sustained, how, by whom and when) and developing and implementing strategies specifically to enhance sustainability. In this regard, the checklist in Fig. 1 can be helpful. Furthermore, monitoring and evaluating sustainability objectives and strategies through time and at all levels of the organized sports setting is important to make adjustments when necessary.

As mentioned previously, program adaptation was an important factor supporting long-term sustainability of programs according to NSFs and sports clubs. Changes to programs were made to align the program with the needs of the (previously) inactive target group and sports clubs, but changes were also made due to the availability of new knowledge or technologies, new partnerships and decreased financial resources. In the literature, program adaptation is also identified as an important factor for program sustainability and it was a pre-specified factor in our framework [29, 30, 34]. However, changing or removing essential components of programs could lead to non-desired outcomes [34, 44]. Therefore, it is important to identify the core elements of the programs that are critical for the achievement of desired outcomes (i.e. increasing physical activity levels of inactive people) and to further study how program adaptation influences these outcomes.

Finally, the sporting programs were aimed at encouraging inactive people to participate in sport. However, it was difficult to recruit large numbers of inactive people using standard recruitment strategies (e.g. flyers, posters). Although activity levels of participants were based on views of the interviewees, they are in accordance with results of an effectiveness evaluation of the NAPSE sporting programs during the three-year implementation period. Based on these results, it appeared that 0 to 15% of participants of the programs was completely inactive at the start [45]. It is important to keep attracting inactive people by promoting the sporting programs in a fun and non-threatening manner, using promotion channels that are appropriate to the target group [25]. Some NSFs and sports clubs did manage to recruit larger numbers of inactive people by collaborating with organizations (e.g. organizations for older people) or people (e.g. a general practitioner, physiotherapist) that are close to this target group. However, trainers of sports clubs often did not have the knowledge or resources to get in contact with inactive people. By sharing knowledge and good practices between NSFs and sports clubs and by educating trainers of sports clubs on how to recruit and engage inactive people (in collaboration with others), participant numbers can be increased. This will contribute to the long-term sustainability of these kind of programs.

### Comparison of factors with other studies

To our knowledge, this is the first study that examined factors influencing the long-term sustainability of sporting programs aimed at inactive target groups and implemented in the organized sports setting. Some of the retrieved sustainability factors that were common to both NSFs and sports clubs are comparable to those found in the study concerning the implementation period of the NAPSE programs [16] and the research about the sustainability of a health promotion program within sport and recreation organizations [23]. For example, alignment of the program with the sport organization’s mission and activities, the availability of financial resources, the provision of training and education opportunities and supportive partnerships. Presumably, these are the basic factors or conditions for continuation of sporting programs, both in the short and longer term and independent of the implementing (sport) organization. The same is true when comparing the common factors with our theoretical framework, which was based on sustainability research of health promotion programs in a diverse range of settings (e.g. healthcare setting, schools, communities) [16, 23, 28–34, 36–38]. This implies there are generic factors influencing the long-term sustainability of a health promotion program independent of the setting in which it is implemented. However, this study also identified new factors (i.e. the inductively added factors), especially those that were related to the specific aim of the NAPSE programs (e.g. program alignment with needs inactive target group) and the (organizational) setting of sports clubs (e.g. dependence on volunteers). The latter also resulted in different influencing factors between NSFs and sports clubs. Sports clubs are largely run by volunteers and the sports clubs setting is, therefore, quite different from the more professional led NSF setting and the other studied settings (e.g. health care setting, schools, communities). Securing human resources (e.g. availability of professional trainers, committed leader) is for sports clubs (as opposed to NSFs) one of their main challenges when continuing program activities over a long period of time. Furthermore, sports clubs ran into other practical issues, like competing activities in their neighborhood and low participant numbers. In addition, the social and fun aspects of participation are inherent to the sports club nature and are important aspects to include in program design. It is important that these factors specific to sports clubs are taken into account, in particular when the NSF is the developer of the program and receiver of the funding and sports clubs are responsible for implementation and continuation of activities. In this regard, the support that NSFs provided to sports clubs (e.g. the provision of training and education opportunities, knowledge and advice, (promotional) materials) facilitated long-term sustainability of programs at sports clubs. Furthermore, as discussed previously, educating trainers of sports club on how to recruit and engage inactive people (in collaboration with others) can be helpful as well in sustaining these kind of programs. In general, this highlights again that it is important to take into account the particular (implementation) context of a program when considering its long-term sustainability [46, 47].

### Practical implications

Overall, the results of this study can be useful to policy makers, sport practitioners and health professionals in countries with a similar organized sports infrastructure. The results can aid them in the development of strategies to promote long-term sustainability of health promotion programs in general and physical activity promotion programs in particular located within the organized sports setting. As mentioned previously, the checklist in Fig. 1 can be used as guidance for this purpose. The checklist can also be helpful in formulating funding guidelines by funding bodies, so that program developers and implementers pay greater attention to the long-term sustainability of their program from the start and integrate sustainability goals and evaluation of these goals in their plans. Program developers and implementers should, for example, elaborate on how they will consider the needs of the inactive target group and how they are going to secure financial resources for the program after the funding period. In this way, the long-term sustainability of these kind of programs will be enhanced.

### Strengths and limitations of the study

Factors influencing long-term sustainability were assessed six and a half years after the funding period ended and both the views of NSFs and sports clubs were examined. In addition, a diverse sample of sporting programs and sporting organizations were included in this study. These are strengths of this study. However, there are also some limitations. Considering the qualitative nature of the study and the Dutch context, it is not known whether the results are generalizable to sport organizations in other countries, particularly as the organizational structure and interplay between organizations may influence the findings. Sports clubs that discontinued programs were harder to find and were less represented in this study (*n* = 11) in comparison with sports clubs that continued programs (*n* = 17). Also, at the level of the NSF there were fewer programs stopped (*n* = 4) than continued (*n* = 10). This may have resulted in more insight into facilitating factors of long-term sustainability than impeding factors. Furthermore, thematic analysis is a proper method to examine views of NSFs and sports clubs regarding key factors influencing the long-term sustainability of their programs and highlighting similarities and differences between the views of these two groups [42]. However, thematic analysis does not focus on the relative importance of these key factors (e.g. are training and education opportunities more important for long-term sustainability than supportive partnerships?). Nonetheless, when considering all of the common and specific factors (see Table 5 and Fig. 1), the likelihood of successfully continuing program activities is increased. Finally, we did not examine possible interactions between influencing factors. For instance, the availability of supportive partnerships (factor *Broader community environment*) could lead to the availability of more financial resources (factor *Organizational setting NSF/sports club*) or the recruitment of larger numbers of inactive people (*Implementation/continuation* factor). In future studies, the possible interaction between factors should be further explored.

## Conclusions

Notwithstanding these strengths and limitations, this study does add to the knowledge base concerning the long-term sustainability of sporting programs aimed at inactive people in the organized sports setting. The key factors facilitating and impeding the long-term sustainability of programs were identified, highlighting similarities and differences in these factors between NSFs and sports clubs. The results of this study can be used by policy makers, sport practitioners and health professionals in countries with a similar organized sports infrastructure in the development of strategies to promote long-term sustainability of these kind of sporting programs. Furthermore, the results can be used to guide funding guidelines. In future research, it is important to identify the core elements of programs that are critical for the achievement of desired outcomes and to further study how program adaptation influences these outcomes. Further research is needed to determine generalizability of the results to differing organized sports settings in other countries. Future research should also take into account possible interactions between influencing factors. This will further contribute to our understanding of how the long-term sustainability of physical activity promotion programs in particular and health promotion programs in general, can be improved in this setting.

## Supplementary information

**Additional file 1.** Theoretical framework of factors. Presents an overview of factors influencing the sustainability of health promotion programs from previous literature, ordered per main theme and subthemes.

**Additional file 2.** Number of interviews with sports clubs. Presents per sporting program the number of interviews conducted with sports clubs.

**Additional file 3.** Interview guide sports clubs continued. Presents the interview guide that is used to interview representatives of sports clubs that continued providing the sporting program.

## Data Availability

The datasets generated and analyzed during the current study are not publicly available to preserve the privacy of participants but are available from the corresponding author on reasonable request.

## Abbreviations

NAPSE: National Action Plan for Sport and Exercise
NSF: National Sports Federation
