# Supplementary material for: Sporting programs aimed at inactive population groups in the Netherlands: factors influencing their long-term sustainability in the organized sports setting
Source: BMC Sports Sci Med Rehabil. 2019 Nov 19;11:33. doi: 10.1186/s13102-019-0137-5 (PMC6862784; doi:10.1186/s13102-019-0137-5)
Supplement: Supplementary file 2 — Additional file 2. Number of interviews with sports clubs. Presents per sporting program the number of interviews conducted with sports clubs. [file 13102_2019_137_MOESM2_ESM.docx]

**Table: Number of telephone interviews with representatives of sports clubs**

| **NAPSE sporting program** | **Included sports clubs that continued the sporting program (n)** | **Included sports clubs that discontinued the sporting program (n)** |
| --- | --- | --- |
| Start to Run (Yakult Start to Run*) | 1 | 2 |
| Judo in School | 1 | 2 |
| Through Four Days Marches | 1 | 2 |
| Working by Walking | 0 | 1 |
| Trendy Weeks for Masters (Flexible*) | 2 | 1 |
| Fit Hockey | 2 | 0 |
| My Swimming Coach | 0 | 0 |
| Thinking and Doing | 2 | 2 |
| Cycle-Fit (Start2Bike*) | 2 | 1 |
| Cycle & Enjoy Nature | 2 | 0 |
| Trio-Triathlon | 1 | 0 |
| Beach Volleyball | 1 | 0 |
| Cool Moves Volley | 2 | 0 |
| Ultimate Volley Xperience | 0 | 0 |
| **Total** | **17** | **11** |

NAPSE=National Action Plan for Sport and Exercise.

* Current name sporting program.
